# A New Fractionation and Recovery Method of Viral Genomes Based on Nucleic Acid Composition and Structure Using Tandem Column Chromatography

**DOI:** 10.1264/jsme2.ME14174

**Published:** 2015-05-23

**Authors:** Syun-ichi Urayama, Yukari Yoshida-Takashima, Mitsuhiro Yoshida, Yuji Tomaru, Hiromitsu Moriyama, Ken Takai, Takuro Nunoura

**Affiliations:** 1 Research and Development Center for Marine Biosciences, Japan Agency for Marine-Earth Science and Technology (JAMSTEC)2–15 Natsushima-cho, Yokosuka, Kanagawa 237–0061Japan; 2 Department of Subsurface Geobiological Analysis and Research, JAMSTEC2–15 Natsushima-cho, Yokosuka, Kanagawa 237–0061Japan; 3 National Research Institute of Fisheries and Environment of Inland Sea, Fisheries Research Agency2–17–5 Maruishi, Hatsukaichi, Hiroshima 739–0452Japan; 4 Laboratory of Molecular and Cellular Biology, Graduate School of Agriculture, Tokyo University of Agriculture and Technology3–5–8 Saiwaicho, Fuchu, Tokyo 183–8509Japan

**Keywords:** viral ecology, viral genome, column chromatography, nucleic acids

## Abstract

Metagenomic studies have revealed the unexplored diversity of the environmental virosphere. However, most studies are biased towards specific types of viral genomes due to the absence of universal methods to access all viral genome types. In the present study, we established a novel system to efficiently separate single- and double-stranded DNA/RNA viral genomes using hydroxyapatite and cellulose chromatography. This method will allow us to quantitatively and simultaneously access four types of viral genomes and will provide important clues to further understand previously unexplored environmental viral populations and obtain potentially unbiased libraries from environmental viral communities.

Most viruses can be classified into four groups based on the compositions of their genomes: single-stranded DNA (ssDNA), double-stranded DNA (dsDNA), single-stranded RNA (ssRNA), and double-stranded RNA (dsRNA) (13). Viral genome types are also often divided into two molecular structures: linear and closed circular. However, some viral replicon types have a limited distribution and propagation in the host species is restricted. Thus, the majority of viruses in natural environments can be subdivided into six types based on the compositions and structures of their viral genomes: linear ssDNA, linear dsDNA, circular ssDNA, circular dsDNA, linear ssRNA, and linear dsRNA (13).

Viruses are ubiquitous and are the most abundant biological entity on Earth (19). Recent genomic studies using next-generation sequencing technology have revealed the unexpected diversity of environmental viruses (4). Furthermore, developments in environmental virology have provided insights into virus-host interactions in important biological processes such as horizontal gene transfer, microbial diversity, and biogeochemical cycling (24). The overview of environmental viruses has mainly been established from dsDNA viruses studied to date (9, 24). However, several recent studies revealed the abundance and significance of ssDNA (14) and ssRNA viruses (7) as well as the presence of dsRNA virus-like elements (8) in aquatic environments. These findings suggest that the ecological and evolutionary impacts of non-dsDNA viruses are present in many biomes. In spite of the potential importance of non-dsDNA viruses, an efficient metagenomic approach that fully covers diverse environmental viruses with different nucleotide compositions and structures has not yet been established due to the absence of quantitative and unbiased extraction-fractionation methods for environmental viral genomes. In particular, a method for the convenient and quantitative fractionation and recovery of viral nucleic acids is required (5).

Several methods have been applied to the fractionation of nucleic acids including HPLC anion-exchange chromatography (6), methylated albumin-kieselguhr columns (15), silica column chromatography (25), cellulose powder (spin-) column chromatography (16, 17), and hydroxyapatite column chromatography (3). Andrews-Pfannkoch *et al.* (1) recently reported a new fractionation method for the simultaneous purification of both viral RNA and DNA using hydroxyapatite chromatography. This method effectively separates circular ssDNA and dsDNA from the nucleic acid mixture; however, linear ssDNA, ssRNA, and dsRNA being eluted in the same fraction. Alternatively, cellulose powder column chromatography is often used to separate viral dsRNA and ssRNA (11, 16). Although Franklin reported that denatured DNA (possibly linear ssDNA) was eluted predominantly in 15% ethanol on cellulose powder column chromatography (11), the elution behavior of linear ssDNA was not examined in detail. In the present study, we defined the ethanol concentration required to elute viral ssDNA using cellulose column chromatography, and then attempted to establish a convenient and quantitative method for viral nucleic acid separation using sequential chromatography with a hydroxyapatite column and cellulose powder spin-column (Fig. 1).

Escherichia coli phage lambda (NBRC20016) and M13 (DSM 13976) and their hosts (NBRC12713 and NBRC3302) were obtained from NBRC (Kisarazu, Japan) and DSMZ (Braunschweig, Germany). Host strains were grown at 37°C in 891 medium (NBRC). Phages were inoculated into cultures in the exponential growth phase and incubated with host cultures overnight. Genomic lambda (dsDNA) and M13 (circular ssDNA) DNA were extracted using the standard phenol/chloroform method and QIAprep Spin M13 kit (QIAGEN, Netherlands), respectively. Chaetoceros tenuissimus DNA virus 06 (CtenDNAV06) was purified as described previously (20), and genomic DNA was extracted using the standard phenol/chloroform method. The CtenDNAV genome was previously shown to be composed of circular ssDNA (5639 nt), including a partially double-stranded region (875 bp) (20). Purified bacteriophage MS2 RNA was purchased (Roche Diagnostics, Switzerland) and used as the representative of linear ssRNA. Magnaporthe oryzae chrysovirus 1 strain A (MoCV1-A) dsRNA was extracted from purified virus particles as described previously (22). A mixture of 50 or 150 ng of each viral nucleic acid was used for the fractionation and recovery test using hydroxyapatite and cellulose powder column chromatography.

In order to determine the optimal ethanol concentration for viral ssDNA binding and elution from the cellulose spin-column, 50 ng of the M13 circular ssDNA was prepared in 1×STE buffer (50 mM Tris/HCl [pH 6.8], 0.1 M NaCl, and 1 mM EDTA) containing 25% ethanol and applied to a spin-column (0.8 mL Empty Spin Column; Bio-Rad) containing cellulose powder (type D; Advantec, Tokyo, Japan) equilibrated with 1×STE buffer containing 25% ethanol. The spin-column was placed in a 2-mL collection tube and centrifuged at 10,000 rpm for 5 s. Three hundred microliters of 1×STE buffer containing 25% ethanol was added to the spin-column and centrifuged. Nucleic acids in the flow-through fraction were precipitated with ethanol. The column was washed with 600 μL of 1×STE buffer containing 15% ethanol, and 600 μL of 1×STE was then added. The nucleic acids eluted with each of the solutions were precipitated with ethanol and analyzed by agarose gel electrophoresis. M13 circular ssDNA was only observed in the flow-through fraction containing 25% ethanol (Fig. 2A). We also confirmed that linearized M13 ssDNA, denatured plasmid pCR2.1 (New England BioLabs, MA, USA), and CtenDNAV06 DNA were recovered in the flow-through fraction as in the case of the M13 circular ssDNA (data not shown). These results indicated that circular, linear ssDNA, and partially double-stranded circular ssDNA did not bind to the cellulose column with 1×STE buffer containing 25% ethanol. Since ssRNA was previously shown to bind cellulose in the presence of >20% ethanol (16), ssDNA, ssRNA (commonly eluted in 1×STE buffer containing 15% ethanol) and dsRNA (commonly eluted in 1×STE buffer without ethanol) can theoretically be separated using cellulose powder spin-column chromatography.

We constructed a tandem chromatography method to separate and retrieve the four viral genome types. A mixture of four known viral genomes (Table 1, Fig. 2B lane 5) was fractionated into dsDNA and non-dsDNA (ssDNA+RNA) as described by Andrews-Pfannkoch *et al.* (1). Briefly, the virus mixture was diluted to half of the column volume with 0.2 M phosphate buffer. After incubation at 60°C for 10 min, the sample was transferred to a sterile and siliconized standard jacketed Econo Column (Bio-Rad, Hercules, CA, USA) containing DNA grade Bio-Gel HTP hydroxyapatite (Bio-Rad) equilibrated with 0.2 M phosphate buffer at 60°C. After being incubated for 30 min, the initial flowthrough was collected. The column was washed with six column volumes of 0.2 M phosphate buffer and then eluted with six column volumes of 1.0 M phosphate buffer. The 0.2 M phosphate buffer flow-through fraction was combined with the initial flowthrough. Each fraction was mixed with an equal volume of phenol-chloroform-isoamyl alcohol (25:24:1 [v/v/v]) and the supernatant was desalted using Amicon Ultra Centrifugal Filter Devices (30,000 MWCO; Millipore, Billerica MA). dsDNA in the 1.0 M phosphate buffer flowthrough fraction was precipitated with ethanol.

The 0.2 M phosphate buffer flowthrough fraction (containing ssDNA+RNA) was fractionated into ssDNA, ssRNA, and dsRNA fractions using a cellulose powder spin-column. The desalted sample was diluted with 1×STE buffer containing 25% ethanol and transferred to a cellulose spin-column. After stepwise elution with 1×STE buffer containing 25% ethanol, 15% ethanol, and finally without ethanol, the resultant fractions were analyzed by agarose gel electrophoresis.

Agarose gel electrophoresis showed that all four viral nucleic acid types were efficiently recovered (Fig. 2B lanes 6–9). Lambda dsDNA was only detected in the 1.0 M phosphate buffer fraction after hydroxyapatite column chromatography (Fig. 2B lane 6). M13 circular ssDNA was detected in the 25% ethanol fraction following cellulose spin-column chromatography (Fig. 2B lane 7). MS2 ssRNA and MoCV1-A dsRNA were detected in the 15% ethanol and 0% ethanol fractions, respectively (Fig. 2B lanes 8 and 9). We also confirmed that Csp03RNAV ssRNA (21) and Alternaria alternate virus-1 dsRNA (2) were eluted in the appropriate fractions as in the case of MS2 ssRNA and MoCV1-A dsRNA, respectively (data not shown). The signal intensity of each viral genome was estimated to be identical before and after chromatographical separation by a semi-quantification analysis with densitometry (Fig. 2B lanes 1–4 and 6–9; Table 1). Neither fragmentation nor contamination with other viral nucleic acids was observed. These results indicated that the four viral genome types were clearly separated and recovered without a quantitative loss using a combination of two types of column chromatography.

We also performed a fractionation of environmental viral genomes using this method (Fig. 2C). Viral particles of coastal water from the quay in JAMSTEC (35°19′9″ N, 139°39′2″ E) were obtained using the iron chloride precipitation method (12). Environmental viral nucleic acids were extracted from the viral concentrate and fractionated by the methods described above. Gel electrophoresis showed that dsDNA was the major component of this viral nucleic acid fraction and ssDNA was also detected (Fig. 2C lanes 1 and 2). However, ssRNA and dsRNA were not identified by gel electrophoresis (Fig. 2C lane 3 and 4). ssRNA was not detected even with the Qubit RNA assay kit (detection limit >5 ng per assay) (Invitrogen, CA, USA) (data not shown). The nucleic acid types eluted in the dsDNA and ssDNA fractions were confirmed by the digestion test using DNA-specific DNaseI (Takara) and ssDNA-specific S1 nuclease (Invitrogen) (data not shown).

This is the first study to show the successful fractionation and full recovery of four viral nucleic acid types. A tandem hydroxyapatite and cellulose spin-column chromatography method may enable us to obtain most of the four viral nucleic acid types without enzymatic digestion treatments. Since enzyme digestion markedly diminishes the total amount of nucleic acids in extracted viral genomes, our method has the advantage of recovering fractionated viral genomes without significant loss from small amounts of precious samples. Thus, to the best of our knowledge, this is the simplest and most efficient method to separate and recover the four types of viral genomes. This newly established efficient and unbiased viral genome fractionation method will allow us to address previously unexplored viral populations in many virospheres, and will provide an important basis for future unbiased virome investigations.

## Figures and Tables

**Fig. 1:**
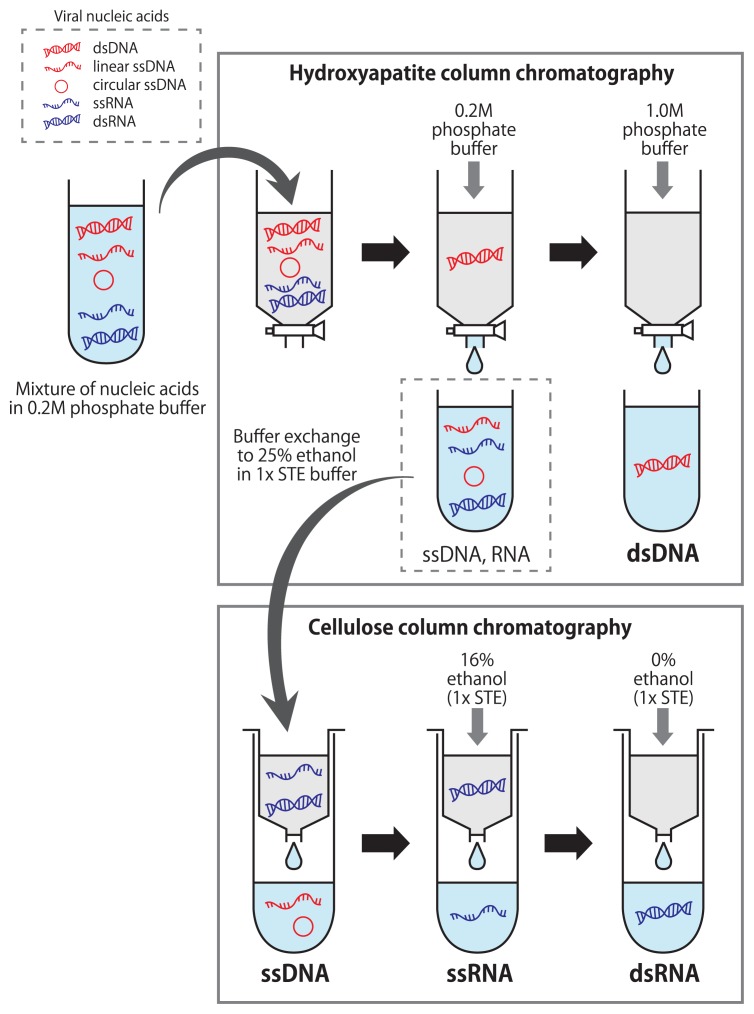
Schematic flow diagram of viral nucleic acid fractionation using tandem hydroxyapatite and cellulose column chromatography.

**Fig. 2:**
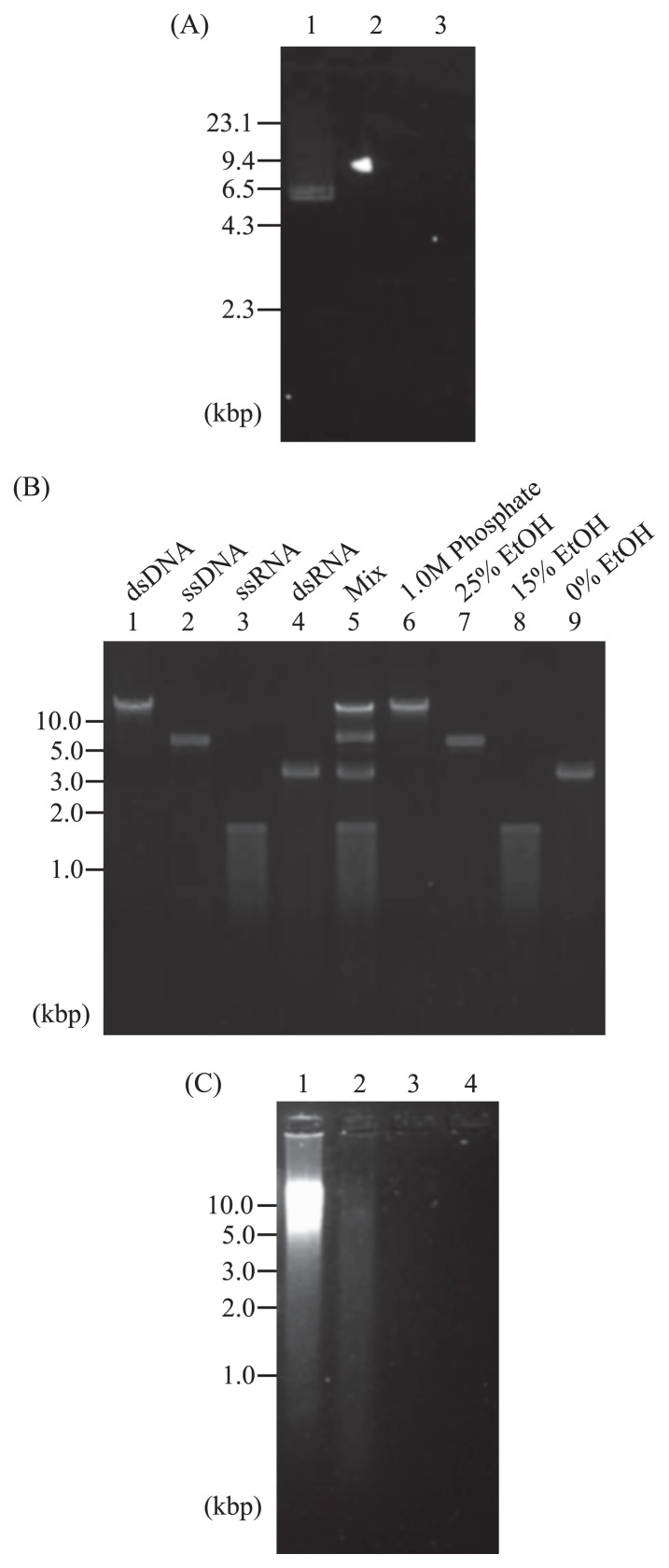
Electrophoretic analysis of fractionated viral nucleic acids. (A) Binding and elution conditions for viral ssDNA on a cellulose column. Fifty nanograms of M13 circular ssDNA was adjusted to 1×STE buffer containing 25% ethanol, and applied to a cellulose spin-column. Lanes 1, 2, and 3 show the flow-through with 1×STE buffer containing 25% ethanol, the fraction eluted with 1×STE containing 15% ethanol, and the fraction eluted with 1×STE, respectively. (B) Separation of four known viral nucleic acid types. In the electropherogram, each of the lanes shows 50 ng of lambda dsDNA (lane 1), M13 circular ssDNA (lane 2), MoCV1-A dsRNA (lane 4), and 150 ng of MS2 ssRNA (lane 3), a mixture of the four viral genomes (50 ng of dsDNA, ssDNA, and dsRNA and 150 ng of ssRNA) (lane 5), the elution fraction of 1.0 M phosphate buffer for a hydroxyapatite column (lane 6), the flowthrough fraction of 25% ethanol in 1×STE buffer of cellulose spin column (lane 7), an elution fraction of 1×STE buffer containing 15% ethanol (lane 8), and an elution fraction eluted with 1×STE (lane 9). (C) Fractionation of environmental viral nucleic acids from 100 mL of coastal sea water from the quay in JAMSTEC (35°19′9″N, 139°39′2″E). Lane 1, the elution fraction of 1.0 M phosphate buffer for a hydroxyapatite column; lane 2, the flow-through fraction of 25% ethanol in 1×STE buffer of cellulose spin column; lane 3, an elution fraction of 1×STE buffer containing 15% ethanol; and lane 4, an elution fraction eluted with 1×STE. Nucleic acids were separated by agarose gel (1%) electrophoresis and visualized with SYBR Gold.

**Table 1:** Characteristics and recovery rates of viruses used in the present study

Virus	Abbreviation	Nucleic Acid	Strandedness	Genome size (s)	References	Recovery rates^a^ (mean ± SD %)
Escherichia coli phage M13	M13	DNA	Single	6,407	(23)	93.2 ± 4.3

Escherichia coli phage λ	λ	DNA	Double	48,502	(18)	93.9 ± 13.7

Escherichia coli phage MS2	MS2	RNA	Single	3,569	(10)	108.6 ± 19.3

				3,554		
				3,250		
Magnaporthe oryzae chrysovirus 1 strain A	MoCV1-A	RNA	Double	3,074	(22)	94.3 ± 3.4
				3,043		
				2,879		

a The intensities of SYBR Gold staining signals were semi-quantified by densitometry using CS-Analyzer ver. 3.0 (ATTO, Japan). Values are the mean ± SD using data from three experiments.
